# The Effects of Smoking on Dental Implant Failure: A Current Literature Update

**DOI:** 10.3390/dj12100311

**Published:** 2024-09-29

**Authors:** Hanna L. Stiller, Josephine Ionfrida, Peer W. Kämmerer, Christian Walter

**Affiliations:** 1 Department of Oral- and Maxillofacial Surgery, University Medical Center Mainz, Augustusplatz 2, 55131 Mainz, Germany; josephine.ionfrida@unimedizin-mainz.de (J.I.); peer.kaemmerer@unimedizin-mainz.de (P.W.K.); walter@mainz-mkg.de (C.W.); 2 Oral and Maxillofacial Surgery, Mediplus Clinic, Haifa-Allee 20, 55128 Mainz, Germany

**Keywords:** smoking, dental implants, implant failure

## Abstract

**Background:** This systematic review assesses the current literature (2020–2024) evaluating the impact of smoking on dental implant failure rates. **Methods:** A non-funded Pubmed database review was conducted according to PRISMA guidelines, and the results were tabulated to extract the study design, patient characteristics, follow-up time, comparison, outcome, and strengths and weaknesses, including risk of bias. This review included 33 studies with 29,519 implants placed in over 18,301 patients. We included prospective and retrospective clinical studies, randomized and non-randomized controlled trials, cohort studies, and observational studies that examined smoking’s effects on implant failure rates. Studies had to classify individuals into two groups, smokers and non-smokers, with at least ten implants. Exclusions included reviews, case reports, experimental studies, guidelines, non-English publications, studies lacking comparative data on failure rates, those excluding smokers, and studies focusing on head and neck cancer patients or specialized implants. **Results:** Our findings indicate a significant correlation in 25 out of 33 studies between smoking and increased implant failure rates, affecting both early and late stages of implant integration and survival as well as revealing a dose–response relationship, with higher daily cigarette consumption significantly increasing the risk of implant failure. **Conclusions:** This review highlights the importance of smoking cessation efforts, patient education, and tailored patient care in dental implantology. Future research should explore the effects of smoking frequency and alternative tobacco products, such as e-cigarettes, aiming to improve success rates among smokers.

## 1. Introduction

Dental implants and the method of implantation have become a widely used techniques for replacing missing teeth, securing bite functionality, and offering aesthetic benefits [1]. Due to continuous technical development and advances in materials, implants now show promising long-term results, making them a preferred choice for tooth replacement [2].

However, the success and failure of dental implants can be influenced by various factors, including systemic health conditions, oral hygiene and periodontal conditions, or lifestyle choices [3]. Literature reports an overall dental implant failure rate of around 5% [4]. However, some studies show rates as low as 1% or as high as 14%, depending on various conditions like patient health, implant location, surgical techniques, follow-up time frame, and criteria used [5].

Among possible risk factors, smoking represents a serious influencing factor, not only affecting oral health but physical health overall [6]. In 2020, the global dataset of the World Health Organization (WHO) reported that 22.3% of adults (aged 15 and above) used some form of tobacco on a current basis, highlighting the pervasive nature of tobacco use [7]. With emerging trends like the consumption of e-cigarettes, the issue of smoking and its impact on dental health is more topical than ever [8]. Previous reviews have investigated the effect of smoking on the failure of dental implants, indicating a correlation between smoking and increased failure rates [9,10]. For example, a comprehensive review reported that failures of implants inserted in smokers are 2.23 times more likely to happen than failures of implants inserted in non-smokers, which does increase the risk of implant failure by 123% [9]. Another review reported that the insertion of implants in smokers significantly affected the failure rates and the risk of postoperative infections as well as marginal bone loss [10]. On the other hand, it is also reported in that study that these results should be interpreted with caution due to the presence of uncontrolled confounding factors in the studies included in the reviews [10].

However, there is a noticeable lack of reviews that address the most current literature covering the past few years. Therefore, this systematic review aims to assess the current impact of smoking on dental implant failure rates and seeks to bridge this gap in the literature by analyzing the findings from studies published from 2020 to 2024. To achieve this, a comprehensive PubMed search was conducted for studies that met the inclusion criteria, focusing on articles that provide quantitative data on implant failure rates among smokers compared to non-smokers. By providing a detailed analysis of recent studies, this review’s objective is to offer valuable insights for both dental professionals and patients, informing clinical decisions and potentially leading to improved implant success rates among smokers. It also aims to highlight the importance of smoking cessation efforts and personalized patient care in the context of dental implantology.

## 2. Materials and Methods

### 2.1. Search Strategies

This review was conducted according to the Preferred Reporting Items for Systematic reviews and Meta-Analyses (PRISMA) Guidelines [5]. This review was previously registered on PROSPERO (ID: CRD42024572472). To acquire the literature related to the topic of this study, an advanced PubMed and Cochrane library search with the terms (dental implant) AND (smoking) AND (implant failure), as well as the MeSH terms (dental implants) AND (smoking), was conducted on 03.03.2024. To focus on the current literature and respond to recent findings, a time restriction was established that included publications from 2020 to 2024.

### 2.2. Inclusion and Exclusion Criteria

For this systematic review, we reviewed prospective and retrospective clinical studies, including randomized and non-randomized controlled trials, cohort studies, and observational studies that investigated the effects of smoking on dental implant failure rates. In addition, we also included studies that compared different groups of individuals (such as young versus old, for example) or different interventions but reported outcomes for the group of smokers versus nonsmokers.

We only included human studies and studies that classified individuals into two groups, smokers and non-smokers, comprising at least a total of ten implants.

We excluded reviews, case reports, experimental studies (in vitro and in vivo), guidelines/recommendation papers, comments, and ongoing clinical trials. Furthermore, publications that were not written in English were excluded, as well as studies that lacked information on dental implant failure rates for smokers versus non-smokers and studies that excluded smokers or did not assess smoking status. Studies that evaluated the success rates of implants placed by students were also excluded. Beyond that, we excluded studies that solely evaluated dental implant failures in head and neck cancer patients or studies that evaluated implants placed in free flaps, failed implant sites, and zygoma and mini dental implants.

### 2.3. Study Selection and Data Extraction

**Figure 1:**
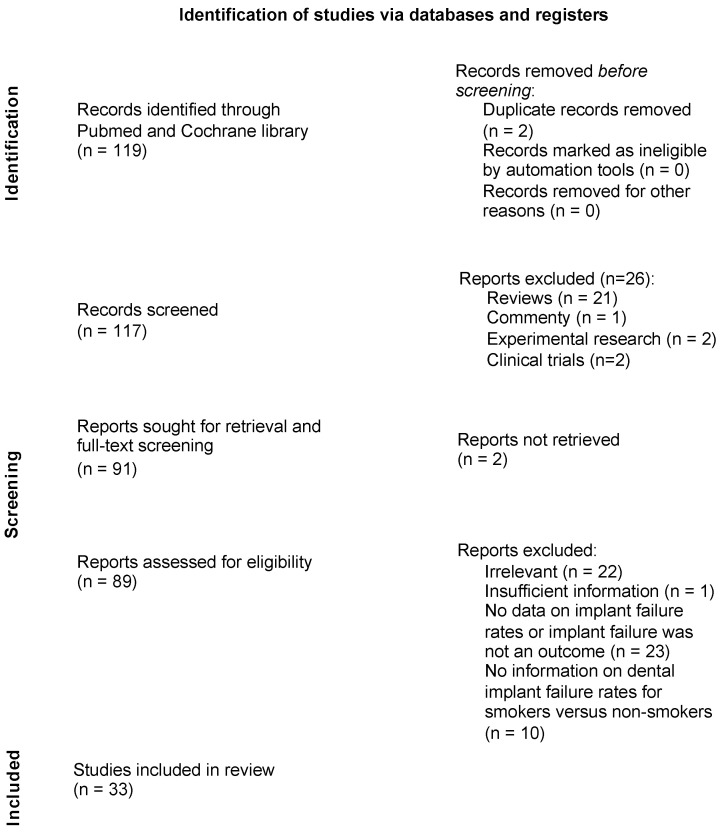
Literature selection process according to the PRISMA flow diagram [5].

The articles were independently assessed for relevance by HLS and JI. In case of any discrepancies, the specific papers were discussed with CW to make a final decision regarding the inclusion (Figure 1). Therefore, the studies were reviewed according to the PICO scheme of evidence-based medicine to clarify the PICO Question: How does smoking impact dental implant outcomes, addressing both the presence of smoking and its intensity as factors? We systematically tabulated the intervention characteristics of each study and compared these against our predefined inclusion criteria. We defined P (patients/population) as patients undergoing dental implant procedures, I (intervention) as smoking, C (comparison) as non-smoking patients, and O (outcome) as rates of dental implant failures.

The data extraction for the included studies was conducted by the authors. Specifically, the following data were extracted: study design, patient characteristics intervention (how many implants were placed), follow-up time, comparison of the investigated factors (implant failure and smoking as a risk factor), conclusion/outcome of the study (is there a connection between implant failure and smoking), and points of strength and weakness including risk of bias of the included studies. In the case of missing or unclear information, we contacted the authors to obtain the missing data, and we did not convert any data. The effect measures to investigate the outcomes included the mean difference, odds ratio, and hazard ratio of the included studies. Implant failure was defined as loss of implant. To explore possible heterogeneity among the studies, a subgroup analysis was conducted based on key variables such as number of implants placed, gender distribution, the distribution among smokers vs. non-smokers, and intervention type.

### 2.4. Risk-of-Bias Analysis

**Figure 2:**
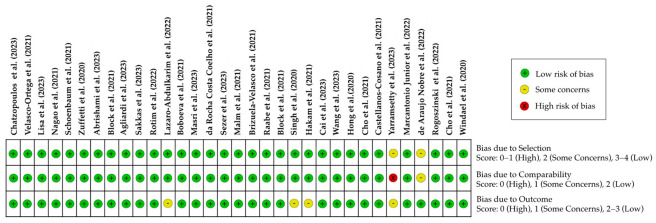
Quality assessment and risk-of-bias analysis based on the Newcastle–Ottawa Scale of the included studies [4,11,12,13,14,15,16,17,18,19,20,21,22,23,24,25,26,27,28,29,30,31,32,33,34,35,36,37,38,39,40,41].

The risk-of-bias analysis of the included studies was conducted by HLS and JI based on the Newcastle–Ottawa Scale. In case of any discrepancies, the results were again discussed with CW to find consensus. The NOS scale evaluates scientific cohort and case-control studies in three categories: selection, comparability, and outcome. The NOS scale contains eight items. For one item, the maximum score is one point (star). Only for the item of comparability is it possible to score 2 points. This leads to a maximum score of nine points. The scoring system can also be found in Figure 2.

### 2.5. Meta-Analysis

Due to heterogeneity in the study designs, such as retrospective cohort studies, prospective analyses, and multi-center studies, the inconsistent comparison groups (for example, non-smokers vs. smokers or heavy smokers vs. light smokers), the inconsistent definitions of outcomes, the varying follow-up times, the investigation of confounding variables in only some studies, and the lack of consistency effect measures and sizes like confidence intervals, a meta-analysis was not performed. Instead, we performed a systematic review that qualitatively synthesizes the findings.

## 3. Results

The literature selection process can be found in Figure 1. The literature search resulted in 564 results in total. Time restriction led to 117 scientific papers, which were then extensively examined and full text-screened for the following defined inclusion and exclusion criteria. First, the abstracts and full texts of the initial 117 results were screened to match the defined inclusion and exclusion criteria. A total of 21 results were reviews, 2 results were experimental studies, 1 study was a comment, and 2 studies were ongoing clinical trials. Two full texts could not be retrieved. A total of 22 results were excluded and labeled as irrelevant because they did not match the inclusion criteria. More precisely, six of these results were excluded because the articles were not written in English, three results because they only evaluated zygoma and mini dental implants, four results had smoking as an exclusion criterion or did not assess smoking status, four results solely evaluated implant success in head and neck cancer patients, two results concerned student-placed implants, and three results only studied implants in failed implant sites or free flaps. Furthermore, we excluded 23 articles that did not have any data on implant failure or where implant failure was not an outcome and 10 results that lacked information on dental implant failure rates for smokers versus non-smokers. We had to exclude one study because the published data were inaccurate. Ultimately, 33 studies were included in this review (Figure 1).The quality assessment and the risk-of-bias analysis can be found in Figure 2. One of the included studies showed a high risk of bias, while four studies did show some concerns about quality and risk of bias. The remaining 28 studies showed a low risk of bias and a high quality (NOS score of 7–8).

Overall results can be found in Table 1. In total, this review evaluated the implant failure rates of 29,519 implants placed in more than 18,301 patients, with sample sizes varying from 22 patients [11] up to 4247 patients [12]. Most of the studies had a retrospective design. Four of the 32 included studies were multi-centered studies [13,14,15,16]. The follow-up times from the studies that disclosed their follow-up periods differed between 1 [17] and up to 22 years [12]. All studies were published between 2020 and 2023. Table 1 demonstrates additional information on the included studies.

Most of the studies found a significant association between smoking and increased dental failure rates. Out of the 33 included studies, only 8 studies did not find a statistically significant association between smoking and increased overall implant failure rates [15,16,18,19,20,21,22,23]. These 8 studies evaluated 11,864 implants in 5475 patients, which is about 1/3 of the evaluated implants and patients.

While closely checking for early failure rates, 7 studies evaluated the rates of early implant failure (EIF) with varying definitions of the term “early”. The time frames ranged from 3 to 4 months [18] up until 12 months [24], or until loading [25]. While there are studies that did not find any correlation between EIF and smoking [18,20], one study found that smokers were 2.14 times more likely to have early implant failure than nonsmokers (OR 2.140, *p* < 0.001) [26]. This finding aligns with two other studies who support this thesis with similar results [25,27]. Another study [24] also found EIF to be higher among smokers (*p* = 0.003). It described increasing odds of EIF of 1.049 for each additional pack-year (*p* < 0.001). Nagao et al. [14] found an OR of 2.07 in smokers compared to never-smokers (OR 2.07) for early implant and an OR for late implant loss of 1.48.

When comparing different smoking frequencies, one study discovered that the risk of implant loss multiplied by 18.3 for smokers with more than 10 cigarettes per day compared to non-smokers, with an overall decrease in implant survival time by 4.2% in smokers vs. non-smokers (*p* = 0.017) [28]. This finding is supported by the similar discovery that heavy smokers (>10 pack years) are at greater risk for failure during implant service (HR 1.81, *p* = 0.039) [12].

Long-term follow-up studies highlight the excellent long-term survival rates of dental implants, with 94% after 15 years [12]. However, when evaluating long follow-up periods, smoking is a crucial factor for implant failure. In one study, smoking represented the major risk factor jeopardizing the survival rate (hazard ratio of 36.35 compared to non-smokers, *p* = 0.001) [29]. When breaking down the probability of implant failure rates by years, one study reported that smoking significantly increased the probability of implant failure in years 1–4 (OR 5.35) as well as after 4 years (OR 4.66) [30].

One included study was particularly large, evaluating the outcome of 1420 dental implants in 826 patients, and the authors found that smoking was associated with the highest proportion of dental implant failures (*p* < 0.05) compared to other medical risk factors, such as diabetes [31]. This finding is supported by another study that evaluated 1820 implants in 771 patients and came to the conclusion that implant failure is significantly higher in smokers than in non-smokers (13.5% vs. 4.4%; *p* = 0.027) and that smokers had a 5.2 times greater risk of implant failure than non-smokers [32].

Several studies of this review have investigated the brand or implant system effects on dental implant failure rates. Eight studies did not find significant differences in implant survival or failure rates between different implant brands [18,19,23,24,26,33,34,35]. Only Lazaro-Abdulkarim et al. [22] found that two of the five implant brands (*p* = 0.021, *p* = 0.024) investigated significantly reduced the risk of implant failure compared to the reference brand. The remaining studies did not investigate brand effects. Most studies included in this review have not explored the possible effect of different restoration types on implant success. The authors of two of the thirty-three included studies wanted to investigate this effect but did not have enough implant losses or only one experimental group with losses at all; therefore, it was not possible to evaluate this effect [28,29]. Nevertheless, one study did find a significant association between the type of prosthesis and failed implants (*p* < 0.05) [36].

## 4. Discussion

This review aimed to examine the current literature and emerging evidence on smoking and dental implant failure. The evidence collected from the systematic review underscores the significant impact of smoking as a critical risk factor for dental implant failure, with a consistent association between smoking and increased implant failure rates observed across multiple studies (Table 1). This aligns with the findings of previous reviews and meta-analyses, for example, Mustapha et al. evaluated studies between 1993 and 2021 [9]. Not only does smoking increase the rate of EIF, but also that of late implant failures [14]. This finding suggests and supports the scientific thesis that smoking does have adverse effects on the initial healing process and implant integration, likely due to the damaging effects of smoking on osteogenesis and angiogenesis, leading to impaired wound healing [26,27]. This association highlights the critical need for considering smoking status in the planning and management of dental implants, emphasizing the role of patient education on smoking cessation, particularly in the early stages of implantation but also in later stages. Furthermore, it reinforces the need for dental professionals to consider these factors when evaluating candidates for dental implants.

In addition, this review provides insights into the importance of distinguishing between different smoking intensities and frequencies. Brizuela-Velasco et al. [22] and French et al. [6] report a dose–response relationship, with higher daily cigarette consumption significantly increasing the risk of implant failure. Unfortunately, the frequency of smoking is a factor that was not commonly assessed in the included studies. In conclusion, these findings show that not only a complete smoking cessation but also a reduction in daily consumption can have a positive effect on implant failure rates, motivating patients who are not able to quit entirely to reduce their daily consumption. It would be interesting for future research to further address this factor.

Long-term follow-up studies underscore the lasting effects of smoking on implant survival, even with the overall high survival rates of dental implants, positioning smoking as a significant risk factor for failure over time [17,29]. Furthermore, findings from large sample studies indicate that smoking is a more significant risk factor for implant failure than other common health risk factors, such as diabetes [31]. These findings underscore the need for ongoing counseling support and intervention for smoking patients, suggesting that smoking reduction and cessation should be a key component in the long-term care plan for patients receiving dental implants. This should motivate dental professionals to address smoking and help patients understand how their smoking habits may influence the treatment outcome.

Eight of the included studies did not find an association between smoking and dental implant failures [15,16,18,19,20,21,22,23]. It is important to note several limitations in a few of these studies that may have contributed to their findings. For example, Abrishami et al. (2023) [18] only included a small number of smokers (2.2%) in their study population, and Sakkas et al. [20] had the proportion of implants placed in smokers underrepresented at follow-up. Zuffetti et al. [16] only investigated a reduced number of lost implants (7/254 implants, four smokers), which could have undermined the power of the statistical analysis. However, there are also studies like Rotim et al. [21] that did include many smokers (n = 224) and still did not find a statistically relevant association.

Overall, the findings of this review suggest that, while brand effects are not universally critical, they may be significant in specific clinical contexts, as demonstrated by the findings of Lazaro-Abdulkarim et al. [22]. Furthermore, when focusing on the effects of different restoration types on implant success and failure, only three studies [28,29,36] investigated, and only one study showed, a significant association between the type of prosthesis and failed implants [36], suggesting that different restoration types could play a role in implant success. More consistent and controlled studies are needed in the future to draw conclusive links, especially for high-risk populations like smokers.

It is important to note that, unfortunately, there were not any studies matching the inclusion criteria that evaluated the impact of e-cigarette use on dental implant failure rates. Further research concerning this topic is therefore much needed.

Another aspect to take into consideration is that tobacco use is one of the most significant risk factors in the development and progression of periodontal disease [43]. History of periodontitis is considered a preponderant risk factor in determining the possible development of severe peri-implant complications [44]. The implications of these findings suggest that a thorough periodontal assessment and history should be integral in the treatment planning of dental implants to mitigate potential long-term complications.

Limitations of this systematic review include publication bias, which can skew the overall findings, heterogeneity among studies, which can complicate the comparison, and differing levels of study quality. Furthermore, this review relies on a literature search of only one database, possibly limiting the number of results.

## 5. Conclusions

In conclusion, this systematic review highlights the critical impact of smoking on the success of dental implants, affecting both the early and late stages of implant integration and survival. Beyond that, this review underscores the need for comprehensive patient assessments, including smoking habits, as part of the planning process of dental implantation. Dental professionals should prioritize counseling and support in smoking reduction and cessation as integral parts of implant treatment. Future research should aim to further elucidate the impact of smoking frequency and explore effective strategies as well as intervention strategies for mitigating the risks for smokers undergoing implant therapy. Longitudinal and interventional studies would be helpful to explore effective strategies for smoking cessation among potential implant patients over a prolonged period. Additionally, investigations focusing on the impact of e-cigarettes and other emerging tobacco products on dental implant outcomes are needed to address the evolving landscape of smoking habits. By integrating findings from a broader range of studies, this review provides a comprehensive and up-to-date perspective on the detrimental effects of smoking on current dental implant success, guiding clinical practice and future research in this area.

## Figures and Tables

**Table 1:** Included studies evaluating smoking as a factor for dental implant failure (M = male; F = female).

Study	Patients	Intervention	Comparison	Conclusion	Association Smoking Implant Loss	Points of Strength	Points of Weakness	Brand Effect (BE) and Restoration Type Effect (RE)
Chatzopoulos et al. (2023) [4]	Not specified	553 implants (278 M/275 F)	Implant failure and patient-related risk factors	Tobacco use (*p* < 0.001) was significantly associated with implant failure	Yes	Many implants investigated, multivariate analysis	Retrospective design, low number of implant failures overall	Brand and restoration type effect not assessed
Velasco-Ortega et al. (2021) [11]	22 patients (10 M/12 F)	198 implants follow-up for 84.2 ± 4.9 months	Loss of implant and risk factors	Loss of implants was significant in patients who smoked up to 10 cigarettes, compared to non-smokers *p* = 0.00104	Yes	Smoking frequency assessed	Excluded smokers >10 cigarettes/day all smoking patients were male small sample size	Brand and restoration type effect not assessed
French et al. (2021) [12]	4247 patients (1852 M/2395 F)	10 871 implants follow-up up to 22.2 years (mean = 4.5 ± 4.2)	Implant failure and potential risk factors	Heavy smokers are at greater risk for failure during implant service (HR 1.81 with 95% CI (1.03, 3.17) and *p* = 0.039	Yes	Long-term, large-scale analysis, multivariate analysis	Retrospective design	Brand and restoration type effect not assessed
Lisa et al. (2023) [13]	107 patients (52 M/55 F)	191 implants 141 sinuses grafted retrospective study	Implant failure following maxillary sinus floor augmentation and risk factors	The risk of implant failure was raised by smoking (OR = 5.8; *p* = 0.012)	Yes	Multicentered	Small sample size	Brand and restoration type effect not assessed
Nagao et al. (2021) [14]	1966 patients (1195 M/771 F)	5052 implants nation wide survey	Early and late implant failure and smoking	Smoking and pack-years were significant factors for total implant loss, OR for smokers compared with never smokers was 2.07 (95% CI 1.19–3.62) for early implant loss and 1.48 (95% CI 0.92–2.37) for late implant loss	Yes	Multicenter study, large sample size, nationwide data, multivariate Analysis	Retrospectively collected secondary data, more detailed information was not collected due to possible biases by non-standardization of the evaluation in this multi-centre study	Brand and restoration type effect not assessed
Schoenbaum et al. (2021) [15]	378 patients (181 M/197 F)	835 implants retrospective, multicenter cohort study mean follow-up of 23.1 months	Dental implant failure and risk factors	Smoking failed to show a statistically significant increase in failure rates.	No	Multicentered, multivariate analysis, three time points	Limited granularity of the patient-level systemic conditions available in private clinical settings	Brand and restoration type effect not assessed
Zuffetti et al. (2020) [16]	174 patients (99 M/75 F)	254 short implants retrospective multicenter study follow-up 3–5 years	Success rate of short implants and potential risk factors	No statistical correlation was found between smoking habit and implant failure	No	Long follow-up time, multicentered	Reduced number of implants lost (7/254 implants, four smokers) could have undermined the power of statistical analysis	Brand and restoration type effect not assessed
Abrishami et al. (2023) [18]	983 patients (428 M/555 F)	983 implants observational study follow-up up to 38 months	Rate of early implant failure and contributing factors	No significant correlation between early failure and smoking habits *p* = 0.316	No	Large sample size	Small number of smokers (2.2%) in study population, retrospective therefore relying on available data from archived records	BT found but not significant (*p* = 0.066), RE not mentioned
Block et al. (2021) [17]	220 patients (83 M/137 F)	Retrospective cohort study 1–12 year follow-up	Implant failure and risk factors	Smoking status is significantly related to implant survival, time to implant removal in smoker vs. non-smoker: HR 2.2 (95% CI: 0.9–5.1) *p* = 0.08	Yes	Multivariate analysis, long follow-up time	Total implant number not mentioned, retrospective data with possible selection bias as well as a nonrandom distribution of patients who are lost to follow-up, single practitioner	Brand and restoration type effect not assessed
Agliardi et al. (2023) [19]	173 patients (80 M/93 F)	692 implants retrospective cohort study 12–15 years follow-up	Implant failure of immediate fixed prostheses supported by two axial and two tilted implants and risk factors	Smoking was not a significant risk factor for implant failure (HR = 0.551), *p* > 0.444	No	Long-term follow-up	Single center, lost-to-follow-up rate of 24% at 15 years (potentially resulting in overestimation of implant success rate)	No difference between brands (*p* > 0.10), CAD-CAM titanium frame-work and acrylic teeth, RE not assessed
Sakkas et al. (2023) [20]	292 patients (275 M/17 F)	466 implants retrospective cohort study 5-year follow-up	Implant failure and impact of clinical variables	Significant correlation of smoking with early implant failure was not detected in this study *p* > 0.999	No	Multivariate analysis, large implant collective	Gender distribution, quite old patient data set, proportion of implants placed in smokers was underrepresented at follow-up, absence of a control group	Brand and restoration type effect not assessed
Rotim et al. (2022) [21]	670 patients	1260 implants follow-up 5 to 10 years	Implant failure and effect of local and systemic factors	No significant differences in dental implant failure between smokers and non-smokers *p* = 0.3587	No	Large sample size, inclusion of many smokers (*n* = 224), long follow-up time	No registration of smoking frequency, no information on gender distribution	Brand and restoration type effect not assessed
Lazaro-Abdulkarim et al. (2022) [22]	1510 patients (720 M/790 F)	4842 implants retrospective cohort study	Failure of dental implants and associated risk factors	No statistically significant differences were found in failure rates among smokers and periodontally compromised patients *p* < 0.05	No	Large sample size, many implants investigated	Most patients were nonsmokers (*n* = 1256), lack of assessment of the quantity and quality of tobacco intake, short follow-up time	Yes, 2/5 brands significantly reduced the risk of implant failure (*p* = 0.021, *p* = 0.024) compared to reference brand, RE not assessed
Boboeva et al. (2021) [23]	1295 patients (584 M/711 F)	2532 implants Retrospective cohort study follow-up 1 to 11 years	Dental implant loss and contributing factors	Smokers did not a statistically significant increased HR compared to nonsmokers in relation to implant failure	No	Large sample size, many implants analyzed, compared old vs. young patients	Retrospective design	Brand was not a significant HR in relation to implant failure, RE not assessed
Masri et al. (2023) [24]	271 patients	751 implants (287 M/464 F) retrospective cohort study	Early implant failure following sinus augmentation and risk factors	EIF was found to be higher among smokers (χ2 (1) = 8.74, *p* = 0.003), among smokers the odds of EIF for each pack year were 1.049 times higher (*p* < 0.001)	Yes	Multivariate analysis, many implants analyzed	Retrospective nature and multiple operators, extreme cases with exceptional systemic or local conditions	Brand was not statistically significant (*p* > 0.05), RE was not assessed
da Rocha Costa Coelho et al. (2021) [25]	594 patients (145 M/449F)	2537 implants retrospective study	Early implant failure and contributing factors	Smoking habits were significantly associated with early implant failure (OR = 2.54; 95% CI (1.00,6.47) and *p* = 0.05)	Yes	Large sample size	Variety of surgeons and systems and missing data in the health charts, unknown follow-up time	Brand and restoration type effect not assessed
Sezer et al. (2023) [26]	1228 patients (582 M/646 F)	4841 implants retrospective study	Rate of early implant failure and contributing factors	Smokers were 2.14 times more likely to have early implant failure than nonsmokers (OR (95% CI): 2.140 (1.438–3.184)) *p* < 0.001	Yes	Large sample size, multivariate analysis	Retrospective design	Implant system (brand) did not have a statistically significant effect (*p* > 0.10), RE not assessed
Malm et al. (2021) [27]	816 patients (402 M/414 F)	4821 implants retrospective case–control study	Early implant failures and contributing factors	Smoking was significantly correlated to early implant failure *p* < 0.05, OR: 2.32 (1.62, 3.32)	Yes	Multivariate analysis, large sample size and many implants placed	Retrospective design so missing data could not be analyzed	Brand and restoration type effect not assessed
Brizuela-Velasco et al. (2021) [28]	110 patients	297 implants (145 M/152 F) retrospective cohort study follow-up 6 years	Implant failure and related factors	Survival time decreased by 4.2% in smokers vs. non- smokers *p* = 0.017, risk of loss multiplied by 18.3 for smokers with more than 10 cigarettes per day	Yes	Long follow-up time	Small number of smokers (*n* = 60) compared to non-smokers (*n* = 231), no overall gender distribution only implant-gender distribution	BE not assessed, RE could not be assessed due to no losses in the experimental group
Raabe et al. (2021) [29]	55 patients (18 M/37 F)	74 implants mean follow-up of 9.1 years (range 4.6–18.2 years)	Implant failure and risk factors	Smoking was the only significant factor jeopardizing the survival rate (hazard ratio of 36.35 compared to non-smokers, *p* = 0.001)	Yes	Long follow-up time in individuals (up to 18 years)	Sample of smokers was very small, various follow-up periods	BE not assessed, RE could not be assessed (only one experimental group with implant losses)
Block et al. (2021) [30]	224 patients (105 M/119 F)	Retrospective case-controlled study	Implant failure and associated factors	Smoking significantly increased the probability of implant failure Year 1–4: aOR 5.35; 95% CI, 1.15 to 25.01), after 4 years: aOR 4.66; 95% CI, 1.45 to 14.99)	Yes	Subdivision into years for detailed factor analysis	No exact number of placed implants only failures, lack of information concerning the experience level of 40% of the patients	Brand and restoration type effect not assessed
Singh et al. (2020) [31]	826 patients (516 M/832 F)	1420 implants retrospective cohort study	Dental implants failures and risk factors	Maximum dental implant failures were seen with smoking (*p* < 0.05)	Yes	Large sample size, many implants	Univariate analysis, retrospective design	Brand and restoration type effect not assessed
Hakam et al. (2021) [32]	771 patients (246 M/425 F)	1.820 implants retrospective clinical study	Implant failure and contributing factors	Implant failure was significantly higher in smokers than in non-smokers (13.5% vs. 4.4%; *p* = 0.027) smokers with 5.2 times greater risk of implant failure than non-smokers	Yes	Many implants analyzed, large sample size	Retrospective design no follow-up time	Brand and restoration type effect not assessed
Cai et al. (2023) [33]	119 patients	642 implants (388 in M/254 in F) (146 prostheses) retrospective cohort study follow-up period from 2 to 7 years	Clinical outcomes of immediate loaded fixed complete dentures and risk factors	Smokers had a significantly lower survival rate than non-smokers (odds ratio: 6.880, *p* = 0.013)	Yes	Long-term follow-up, many implants investigated	Retrospective design, unicenter study, no registration of smoking frequency	No significant differences in implant survival based on implant system (brand) *p* > 0.05, RE not assessed
Wang et al. (2023) [34]	123 patients (72 M/51 F)	123 implants retrospective cohort study 3- to 12-year follow-up	Implant failure after lateral sinus floor elevation and contributing factors	Smoking habit significantly jeopardized implant survival (HR: 6.055, *p* = 0.024)	Yes	Multivariate analysis	Retrospective design, small sample size, low overall loss rate of implants might limit the detection on variables	Brand did not influence implant survival (*p* > 0.563), RE not assessed
Hong et al. (2020) [35]	240 patients (134 M/106 F)	399 implants retrospective cohort study mean follow-up of 30.6 ± 12 months	Implant survival and risk factors	Smoking significantly increased the failure rate (hazard ratio, 10.7; *p* = 0.002)	Yes	Long follow-up time, multivariate Analysis	Retrospective design	Implant system (brand) is not significant factor for implant failure (*p* > 0.21), RE not assessed
Castellanos-Cosano et al. (2021) [36]	143 patients	456 implants Retrospective multicentre and cross-sectional cohort study	Implant loss and marginal bone loss and risk factors	Smokers are more likely to lose implants (*p* < 0.05).	Yes	Many implants investigated	Retrospective design	BE was not assessed, significant association between type of prosthesis and failed implants (*p* < 0.05)
Yarramsetty et al. (2023) [37]	80 patients	100 implants retrospective study	Dental implant failures and risk factors	Smoking was linked to the greatest number of dental implant failures *(p*= 0.001)	Yes	More than one implant per patient	Small sample size, uncertain follow-up time, no information on gender distribution	Brand and restoration type effect not assessed
Marcantonio Junior et al. (2022) [38]	58 patients (21 M/37 F)	86 extra-narrow implants retrospective study follow-up for up to eight years	Implant success of extra-narrow implants and influence of risk factors	Correlation between smoking and implant loss, 8× more likely in smokers than non-smokers (*p* = 0.024) (95% CI 1.0–63.9)	Yes	Long-term follow-up	Retrospective design, missing information can lead to information bias, small sample size	Brand and restoration type effect not assessed
de Araujo Nobre et al. (2022) [39]	123 patients (38 M/85 F)	192 implants in immediate function presenting dehiscence, fenestrations or both with All-on-4-concept 10 years follow-up	Cumulative implant survival and success rates in challenging conditions and medical status distribution	Smoking affected implant failure significantly (*p* = 0.019)	Yes	Long-term follow-up frequent follow-ups at 10 days, 2/4/6 months, 1 year, and every 6 months thereafter	Small sample size that disabled inferential analysis lack of stratification for smoking habits, significant difference in age between the sample lost to follow-up and the fully analyzed sample	Brand and restoration type effect not assessed, titanium framework and all-ceramic crowns or acrylic resin prosthetic teeth
Rogoszinski et al. (2022) [40]	284 patients	933 implants (870M/66 F) retrospective cohort study 5 years of follow-up	Implant failure and contributing factors	Current smoking (late failure OR, 1.62; *p* = 0.01) increased the odds of long-term implant failure.	Yes	Long-term follow up, many implants investigated	Gender distribution, patient records only included information regarding continued use of the medication throughout the follow-up period	Brand and restoration type effect not assessed
Cho et al. (2021) [41]	78 patients	104 implants retrospective study 3-year follow-up	Implant failure and contributing factors	Smoking (HR, 5.4; 95% CI, 1.5–20.5; *p* = 0.018) significantly increased the risk of implant failure	Yes	Long follow-up time	Retrospective design with missing parameters because of insufficient records	Brand and restoration type effect not assessed
Windael et al. (2020) [42]	121 patients (48 M/73 F)	453 implants prospective analysis mean follow-up time of 11.38 years	Implant success and long-term effect of smoking	Maxilla showed significant difference of implant success between smokers and non-smokers (*p* = 0.003/*p* = 0.007) hazard of implant loss 5.64× higher in smokers than non-smokers (*p* = 0.003)	Yes	Long follow-up time prospective analysis	No multivariate analysis relatively small population	Brand and restoration type effect not assessed

## Data Availability

The original contributions presented in the study are included in the article, further inquiries can be directed to the corresponding author.

## References

[1] Alghamdi H.S. (2018). Methods to Improve Osseointegration of Dental Implants in Low Quality (Type-IV) Bone: An Overview. J. Funct. Biomater..

[2] Alam M.K., Rahman S.A., Basri R., Sing Yi T.T., Si-Jie J.W., Saha S. (2015). Dental Implants—Perceiving Patients’ Satisfaction in Relation to Clinical and Electromyography Study on Implant Patients. PLoS ONE.

[3] Raikar S., Talukdar P., Kumari S., Panda S.K., Oommen V.M., Prasad A. (2017). Factors Affecting the Survival Rate of Dental Implants: A Retrospective Study. J. Int. Soc. Prev. Community Dent..

[4] Chatzopoulos G.S., Wolff L.F. (2023). Dental implant failure and factors associated with treatment outcome: A retrospective study. J. Stomatol. Oral Maxillofac. Surg..

[5] Page M.J., McKenzie J.E., Bossuyt P.M., Boutron I., Hoffmann T.C., Mulrow C.D., Shamseer L., Tetzlaff J.M., Akl E.A., Brennan S.E. (2021). The PRISMA 2020 statement: An updated guideline for reporting systematic reviews. BMJ.

[6] Beklen A., Sali N., Yavuz M.B. (2022). The impact of smoking on periodontal status and dental caries. Tob. Induc. Dis..

[7] WHO (2021). WHO Global Report on Trends in Prevalence of Tobacco Use 2000–2025.

[8] Huilgol P., Bhatt S.P., Biligowda N., Wright N.C., Wells J.M. (2019). Association of e-cigarette use with oral health: A population-based cross-sectional questionnaire study. J. Public Health.

[9] Mustapha A.D., Salame Z., Chrcanovic B.R. (2021). Smoking and Dental Implants: A Systematic Review and Meta-Analysis. Medicina.

[10] Chrcanovic B.R., Albrektsson T., Wennerberg A. (2015). Smoking and dental implants: A systematic review and meta-analysis. J. Dent..

[11] Velasco-Ortega E., Jimenez-Guerra A., Ortiz-Garcia I., Moreno-Munoz J., Nunez-Marquez E., Cabanillas-Balsera D., Lopez-Lopez J., Monsalve-Guil L. (2021). Immediate Loading of Implants Placed by Guided Surgery in Geriatric Edentulous Mandible Patients. Int. J. Environ. Res. Public Health.

[12] French D., Ofec R., Levin L. (2021). Long term clinical performance of 10 871 dental implants with up to 22 years of follow-up: A cohort study in 4247 patients. Clin. Implant Dent. Relat. Res..

[13] Lisa K., Flore D., Gaetan V.V., Yannick S., Constantinus P. (2023). Survival rate of implants following maxillary sinus floor augmentation using freeze-dried allografts vs bovine derived xenografts: A retrospective multicenter study. J. Stomatol. Oral Maxillofac. Surg..

[14] Nagao T., Fukuta J., Sugai T., Kawana H., Matsuo A., Hamada S., Miura K., Seto K. (2021). Prevalence of early and late oral implant loss among smokers: A nationwide survey in Japan. Int. J. Oral Maxillofac. Surg..

[15] Schoenbaum T.R., Moy P.K., Aghaloo T., Elashoff D. (2021). Risk Factors for Dental Implant Failure in Private Practice: A Multicenter Survival Analysis. Int. J. Oral Maxillofac. Implant..

[16] Zuffetti F., Testarelli L., Bertani P., Vassilopoulos S., Testori T., Guarnieri R. (2020). A Retrospective Multicenter Study on Short Implants With a Laser-Microgrooved Collar (≤7.5 mm) in Posterior Edentulous Areas: Radiographic and Clinical Results up to 3 to 5 Years. J. Oral Maxillofac. Surg..

[17] Block M.S., Christensen B.J. (2021). Porous Bone Increases the Risk of Posterior Mandibular Implant Failure. J. Oral Maxillofac. Surg..

[18] Abrishami M.H., Shiezadeh F., Samieirad S., Mollaei M., MohammadZadeh Mahrokh F., Khosravi F. (2023). Analyzing the Causes and Frequency of Early Dental Implant Failure among Iranians: An Epidemiological Study. Int. J. Dent..

[19] Agliardi E.L., Pozzi A., Romeo D., Del Fabbro M. (2023). Clinical outcomes of full-arch immediate fixed prostheses supported by two axial and two tilted implants: A retrospective cohort study with 12–15 years of follow-up. Clin. Oral Implant. Res..

[20] Sakkas A., Westendorf S., Thiele O.C., Schramm A., Wilde F., Pietzka S. (2023). Prosthetically guided oral implant surgery. A retrospective cohort study evaluating the 5-year surgical outcome. GMS Interdiscip. Plast. Reconstr. Surg. DGPW.

[21] Rotim Z., Pelivan I., Sabol I., Susic M., Catic A., Bosnjak A.P. (2022). The Effect of Local and Systemic Factors on Dental Implant Failure—Analysis of 670 Patients with 1260 Implants. Acta Clin. Croat..

[22] Lazaro-Abdulkarim A., Lazaro D., Salomo-Coll O., Hernandez-Alfaro F., Satorres M., Gargallo-Albiol J. (2022). Failure of Dental Implants and Associated Risk Factors in a University Setting. Int. J. Oral Maxillofac. Implant..

[23] Boboeva O., Kwon T.G., Kim J.W., Lee S.T., Choi S.Y. (2021). Comparing factors affecting dental-implant loss between age groups: A retrospective cohort study. Clin. Implant Dent. Relat. Res..

[24] Masri D., Jonas E., Avishai G., Rosenfeld E., Chaushu L., Chaushu G. (2023). Risk factors contributing to early implant failure following sinus augmentation: A study of a challenging cohort. J. Oral Rehabil..

[25] da Rocha Costa Coelho T., Almeida de Azevedo R., Borges Maia W.W., Nunes Dos Santos J., Ramos Cury P. (2021). Evaluation of the Association of Early Implant Failure With Local, Environmental, and Systemic Factors: A Retrospective Study. J. Oral Maxillofac. Surg..

[26] Sezer T., Soylu E. (2023). COVID-19 as a factor associated with early dental implant failures: A retrospective analysis. Clin. Implant Dent. Relat. Res..

[27] Malm M.O., Jemt T., Stenport V.F. (2021). Patient factors related to early implant failures in the edentulous jaw: A large retrospective case-control study. Clin. Implant Dent. Relat. Res..

[28] Brizuela-Velasco A., Alvarez-Arenal A., Perez-Pevida E., Bellanco-De La Pinta I., De Llanos-Lanchares H., Gonzalez-Gonzalez I., Larrazabal-Moron C. (2021). Logistic Regression Analysis of the Factors Involved in the Failure of Osseointegration and Survival of Dental Implants with an Internal Connection and Machined Collar: A 6-Year Retrospective Cohort Study. Biomed. Res. Int..

[29] Raabe C., Monje A., Abou-Ayash S., Buser D., von Arx T., Chappuis V. (2021). Long-term effectiveness of 6 mm micro-rough implants in various indications: A 4.6- to 18.2-year retrospective study. Clin. Oral Implant. Res..

[30] Block M.S., Christensen B.J., Mercante D.E., Chapple A.G. (2021). What Factors Are Associated With Implant Failure?. J. Oral Maxillofac. Surg..

[31] Singh R., Parihar A.S., Vaibhav V., Kumar K., Singh R., Jerry J.J. (2020). A 10 years retrospective study of assessment of prevalence and risk factors of dental implants failures. J. Fam. Med. Prim. Care.

[32] Hakam A.E., Vila G., Duarte P.M., Mbadu M.P., Ai Angary D.S., Shuwaikan H., Aukhil I., Neiva R., da Silva H.D.P., Chang J. (2021). Effects of different antidepressant classes on dental implant failure: A retrospective clinical study. J. Periodontol..

[33] Cai B., Tong Z., Qian Y., Zhu W., Lu Y., Si M. (2023). Immediate loaded fixed complete dentures supported by implants in patients with a history of periodontitis: A retrospective cohort study of 2 to 7 years. J. Prosthet. Dent..

[34] Wang Z., Zhang J., Li N., Pu R., Wang Y., Yang G. (2023). Survival analysis of implants placed simultaneously with lateral sinus floor elevation in severely atrophic maxilla: A 3- to 12-year retrospective cohort study. Clin. Implant Dent. Relat. Res..

[35] Hong J.Y., Shin E.Y., Herr Y., Chung J.H., Lim H.C., Shin S.I. (2020). Implant survival and risk factor analysis in regenerated bone: Results from a 5-year retrospective study. J. Periodontal Implant Sci..

[36] Castellanos-Cosano L., Carrasco-Garcia A., Corcuera-Flores J.R., Silvestre-Rangil J., Torres-Lagares D., Machuca-Portillo G. (2021). An evaluation of peri-implant marginal bone loss according to implant type, surgical technique and prosthetic rehabilitation: A retrospective multicentre and cross-sectional cohort study. Odontology.

[37] Yarramsetty G.V., Singiri B.M., Vijay K.R., Balaji V.C., Anusha K., Thota R.P. (2023). A Retrospective Analysis to Assess the Reasons for the Failure of Dental Implants. J. Pharm. Bioallied Sci..

[38] Marcantonio Junior E., Sartori I.A.M., Vianna C.P., Rocha R.S., Caldas W., Trojan L.C. (2022). Influence of risk factors on the long-term survival of oral rehabilitation with extra-narrow implants: A retrospective study. J. Appl. Oral Sci..

[39] de Araujo Nobre M., Lopes A., Antunes E. (2022). The 10 Year Outcomes of Implants Inserted with Dehiscence or Fenestrations in the Rehabilitation of Completely Edentulous Jaws with the All-on-4 Concept. J. Clin. Med..

[40] Rogoszinski T., Dazen C., Rekawek P., Coburn J.F., Carr B.R., Boggess W., Chuang S.K., Lee K.C., Panchal N., Ford B.P. (2022). Are proton pump inhibitors associated with implant failure and peri-implantitis?. Oral Surg. Oral Med. Oral Pathol. Oral Radiol..

[41] Cho Y.D., Kim S., Ku Y. (2021). Effectiveness of dental implantation with the partial split-flap technique on vertical guided bone regeneration: A retrospective study. J. Periodontal Implant Sci..

[42] Windael S., Vervaeke S., De Buyser S., De Bruyn H., Collaert B. (2020). The Long-Term Effect of Smoking on 10 Years’ Survival and Success of Dental Implants: A Prospective Analysis of 453 Implants in a Non-University Setting. J. Clin. Med..

[43] Johnson G.K., Guthmiller J.M. (2007). The impact of cigarette smoking on periodontal disease and treatment. Periodontol. 2000.

[44] Serroni M., Borgnakke W.S., Romano L., Balice G., Paolantonio M., Saleh M.H.A., Ravida A. (2024). History of periodontitis as a risk factor for implant failure and incidence of peri-implantitis: A systematic review, meta-analysis, and trial sequential analysis of prospective cohort studies. Clin. Implant Dent. Relat. Res..

